# Sarcoidosis Presenting as Löfgren's Syndrome with Myopathy

**DOI:** 10.1155/2013/125251

**Published:** 2013-04-03

**Authors:** Şenol Kobak, Murat Yalçin, Fidan Sever, Guray Oncel

**Affiliations:** ^1^Department of Rheumatology, Şifa University Faculty of Medicine, Bornova, 35100 Izmir, Turkey; ^2^Department of Chest Diseases, Şifa University Faculty of Medicine, Izmir, Turkey; ^3^Department of Radiology, Şifa University Faculty of Medicine, Izmir, Turkey

## Abstract

A 34-year-old female patient, who had proximal muscle weakness for 8 months, presented with erythema nodosum lesions on the pretibial region in addition to pain, swelling, and movement restriction in both ankles for the last one month. Thoracic CT demonstrated hilar and mediastinal lymphadenopathy. She underwent mediastinoscopic lymph node biopsy; biopsy result was consistent with noncaseating granuloma. Serum angiotensin converting enzyme level and muscle enzymes have been elevated. Muscular MRI and EMG findings were consistent with myositis. Muscle biopsy was done, and myopathy was found. The patient was diagnosed with sarcoidosis, Löfgren's syndrome, and sarcoid myopathy. The patient displayed remarkable clinical and radiological regression after 6-month corticosteroid and MTX therapy.

## 1. Introduction

Sarcoidosis is a systemic granulomatous disease of unknown origin and can involve many tissues and organs in the body. The disease most commonly presents with bilateral hilar lymphadenopathy, lung infiltrations, and skin and ophthalmic lesions [1]. Löfgren's syndrome (LS) is an acute sarcoidosis presentation characterized by arthritis/arthralgia, erythema nodosum (EN), and bilateral hilar lymphadenopathy [2]. Musculoskeletal system involvement may usually be in the form of synovitis of the large joints of lower extremities. Sarcoidosis may involve proximal muscles and may mimic idiopathic inflammatory myositis [3]. Symptomatic muscular disease is rare and seen in 0.5–5% of the cases [4]. It has three different types: chronic myopathy, palpable nodules, and acute myositis [5]. In the present case, we have reported the presence of chronic sarcoid myopathy together with Löfgren's syndrome.

## 2. Case Report

**Figure 1:**
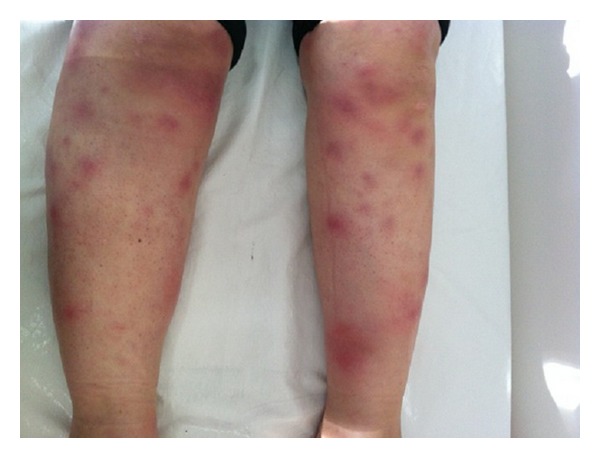
Erythema nodosum lesions on the bilateral pretibial regions.

**Figure 2:**
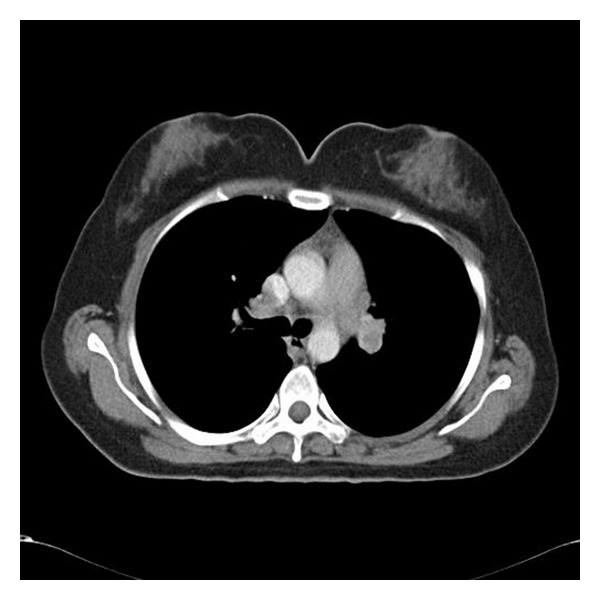
Bilateral hilar lymphadenopathy on thoracic CT.

**Figure 3:**
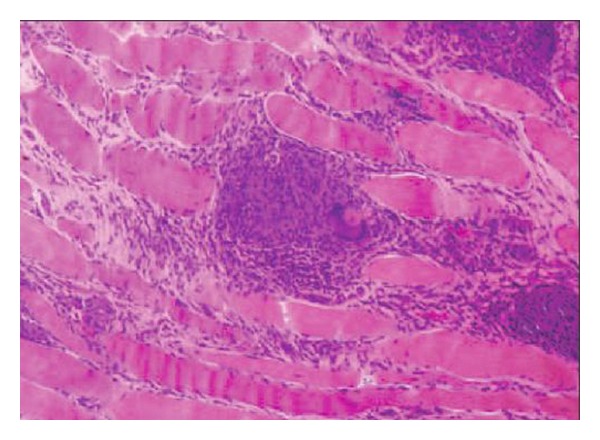
Muscle biopsy of a patient with sarcoid myopathy demonstrates fascicular inflammatory infiltrates with multinucleate giant cells.

A 34-year-old female patient consulted with several physicians for proximal muscle weakness, which had continued and worsened for 8 months. Her complaints became worse despite NSAIDs and she presented to our outpatient clinic of rheumatology because of pain, swelling, and restricted motion in both ankles, as well as painful erythematous lesions on the pretibial region, which started a month ago. On the inspection, erythema nodosum skin lesions were detected on the pretibial region (Figure 1). Musculoskeletal system examination revealed synovitis in both ankles and proximal muscle weakness in the proximal extremities. Results of laboratory analysis were as follows; SGOT: 152 U/L (normal 0–30 U/L), SGPT: 144 U/L (normal 0–40 U/L), alkaline phosphatase: 46 U/L (normal < 90 U/L), LDH: 318 U/L (normal 135–220 U/L), CK: 1213 U/L (normal 26–192 U/L), C-reactive protein (CRP): 8.41 mg/dL (normal 0–0.5 mg/dL), erythrocyte sedimentation rate (ESR): 67 mm/h (normal 0–20 mm/h), TSH: 2.81 uIU/mL (normal 0.27–4.2 uIU), serum angioconverting enzyme (ACE): 78 (normal 0–25), and serum Ca: 10.8 (normal 0–9 mg/dL). Complete blood count showed anemia of chronic disease. On serological analysis, rheumatoid factor (RF) and anti-nuclear antibody (ANA) were negative and complements (C3 and C4) were within the normal ranges. Mild hilar fullness was observed on the chest radiography. Thoracic CT demonstrated multiple bilateral hilar and mediastinal lymphadenopathies, of which the largest one was two centimeters (Figure 2). These findings were considered in favor of sarcoidosis. No pathological finding, except for hepatosteatosis, was detected on the abdominal US and CT. The patient was consulted with the Department of Chest Diseases from which an endobronchial ultrasonographic (EBUS) guided lymph node biopsy was recommended. Subsequently the results from the EBUS found that no endobronchial lesion was detected, and the pathology report revealed normal findings except for patchy histiocytes. Thus, a mediastinoscopic lymph node biopsy was performed, detecting noncaseating granulomas on pathological assessment, suggesting likely sarcoidosis as stated in pathological account. An MRI was performed for proximal muscles of lower extremity and a T2 signal increment was observed in gluteus maximus muscle on the gluteal region. Lower extremity EMG findings were consistent with myositis, in which the spontaneous denervation potentials were prominent. A muscle biopsy was taken indicating typical noncaseating granulomas, perivascular inflammation, and muscle fiber degeneration and regeneration was found (Figure 3). Based on clinical, radiological, and pathological data, the patient was diagnosed with sarcoidosis, sarcoid myopathy, and Löfgren-syndrome. The patient was given corticosteroid (CS) 1 mg/kg/day and methotrexate (MTX) 10 mg/week. Remarkable clinical and laboratory regression was observed during policlinic control visit on the sixth month of treatment. 

## 3. Discussion

Löfgren's syndrome (LS) is an acute sarcoidosis characterized by the triad of erythema nodosum, bilateral hilar lymphadenopathy, and arthritis [6]. The incidence of LS changes among countries and ethnic races. In Europe and USA, sarcoidosis initially presents with LS in 10% of patients [7]. While it is rare in Africans, it appears to be common in young white females and is found to be associated with HLA-B8 and DR3 [8]. Sarcoidosis has generally a good prognosis in cases that present with LS; 90% of patients indicate complete remission within the first two years. Although it is known as a self-limiting syndrome, some cases develop chronic sarcoidosis [9]. The present case also exhibits high serum angiotensin converting enzyme concentration in addition to the classical triad of LS. The patient underwent lymph node biopsy under EBUS guidance, which is used for the diagnosis of lung pathologies and considered to be a reliable method. We failed to find an exact result due to an insufficient amount of biopsy material, therefore a mediastinoscopic lymph node biopsy was performed. A pathological examination of the biopsy material revealed noncaseating granulomas. The patient had muscle weakness together with LS. Analyses indicated elevated muscular enzyme levels and myopathy was determined based on muscular MRI, EMG, and muscle biopsy. 

Sarcoid myopathy was first defined in 1908 in a 17-year-old female patient with lupus pernio, splenomegaly, and multiple nodules in the muscles [10]. Whereas asymptomatic muscle involvement is seen in 25–75% of the patients, symptomatic involvement accounts for only 0.5–5%. Symptomatic myopathy may present in three different forms: chronic myopathy, palpable nodules, or acute myositis [10]. Acute myositis may be the first sign of sarcoidosis and/or a part of the progressive form [11]. Nodular form is quite rare and is characterized by tumor-like lesions in the muscles [12]. It has been reported in only three cases in two large series comprising 1300 sarcoidosis patients. To date, only about sixty cases with nodular sarcoidosis have been reported in the literature. Chronic myopathy, which presents with proximal muscle weakness developed over months and years, is the most common form [13]. It may be accompanied by muscular atrophy, contracture, or pseudo hypertrophy. Muscular pain may begin before muscle weakness. Although rare, distal muscle involvement may be combined with peripheral neuropathy. Diagnosis is made based on elevated muscular enzyme levels, imaging methods (muscular MRI, Ga scintigraphy, and PET), EMG findings, and/or muscle biopsy. It has been explained that noncaseating granulomas infiltrate perimysial connective tissue and cause muscular atrophy and fibrosis on muscle biopsy [14]. However, it is not a condition to see noncaseating granulomas. Presence of different clinical and pathological signs in different cases of sarcoid myopathy raises the thought that the disease has a complex and complicated pathophysiology. While some cases display granuloma formations that cause destruction in muscle fibers, other cases can display only myopathic changes. Immunological studies demonstrate high CD4 T lymphocyte and CD4/CD8 ratio in sarcoid myopathies, whereas there is CD8 T-lymphocyte predominance in polymyositis and in inclusion body myositis [15]. Cellular immune mechanisms play a key role in the pathogenesis of polymyositis [16]. In vitro studies demonstrated that peripheral blood lymphocytes are toxic for myoblasts and fibroblasts. Lymphocytes may lead to muscular damage either directly causing cytolysis in muscular fibers or indirectly over the cytokines. Sarcoid myopathy may share the mechanisms of polymyositis. Presence of inflammatory and degenerative processes without granuloma in the muscles raises the thought that it occurs due to the cytokines released from apart. Therefore, studies have shown increased serum concentrations of IL-1 IL-2 in sarcoidosis patients [17]. Corticosteroids are used for the treatment of sarcoid myopathy. Conflicting results have been reported about therapeutic response. In the present patient case, remarkable clinical, laboratory, and radiological regression was achieved with a CS and MTX combination.

In conclusion, we reported a case with sarcoid myopathy and LS. Sarcoidosis may be a great mimicker of different diseases or may present with idiopathic, inflammatory myositis-like picture as well. New prospective studies are required to illuminate the etiopathogenesis of sarcoid myopathy.
